# Motivation and Barriers for Leisure-Time Physical Activity in Socioeconomically Disadvantaged Women

**DOI:** 10.1371/journal.pone.0147735

**Published:** 2016-01-25

**Authors:** Inês Santos, Kylie Ball, David Crawford, Pedro J. Teixeira

**Affiliations:** 1 Interdisciplinary Center for the Study of Human Performance, Faculty of Human Kinetics, University of Lisbon, Cruz Quebrada, Lisbon, Portugal; 2 Centre for Physical Activity and Nutrition Research, Faculty of Health, Deakin University, Melbourne, Australia; University of Bath, UNITED KINGDOM

## Abstract

**Introduction:**

The aim of this study was to examine cross-sectional and longitudinal associations between motivation and barriers for physical activity, and physical activity behavior in women living in socioeconomic disadvantage. This study also examined whether weight control intentions moderate those associations.

**Methods:**

Data from 1664 women aged 18–46 years was collected at baseline and three-year follow-up as part of the Resilience for Eating and Activity Despite Inequality study. In mail-based surveys, women reported sociodemographic and neighborhood environmental characteristics, intrinsic motivation, goals and perceived family barriers to be active, weight control intentions and leisure-time physical activity (assessed through the IPAQ-L). Linear regression models assessed the association of intrinsic motivation, goals and barriers with physical activity at baseline and follow-up, adjusting for environmental characteristics and also physical activity at baseline (for longitudinal analyses), and the moderating effects of weight control intentions were examined.

**Results:**

Intrinsic motivation and, to a lesser extent, appearance and relaxation goals for being physically active were consistently associated with leisure-time physical activity at baseline and follow-up. Perceived family barriers, health, fitness, weight and stress relief goals were associated with leisure-time physical activity only at baseline. Moderated regression analyses revealed that weight control intentions significantly moderated the association between weight goals and leisure-time physical activity at baseline (β = 0.538, 99% CI = 0.057, 0.990) and between intrinsic motivation and leisure-time physical activity at follow-up (β = 0.666, 99% CI = 0.188, 1.145). For women actively trying to control their weight, intrinsic motivation was significantly associated with leisure-time physical activity at follow-up (β = 0.184, 99% CI = 0.097, 0.313).

**Conclusions:**

Results suggest that, especially in women trying to control their weight, intrinsic motivation plays an important role in sustaining physical activity participation over time. Also, weight goals for being physically active seem to play a role regarding short-term physical activity participation in this particular population. Addressing these motivational features may be important when promoting physical activity participation in women living in socioeconomically disadvantaged neighborhoods.

## Introduction

The health benefits associated with regular participation in physical activity, as well as the positive role of physical activity for successful weight reduction and maintenance, are well documented [1, 2]. Yet, many adults in developed countries fail to meet public health guidelines for recommended levels of physical activity. For instance, 2011 data indicate that, across the World Health Organization (WHO) regions, the frequency of physical inactivity (i.e., not engaging in 30 min of moderate-intensity physical activity on at least 5 days every week or 20 min of vigorous-intensity activity on at least 3 days every week, or a combination of both) varies between 17% in southeast Asia and about 43% in the Americas and the eastern Mediterranean [3]. Of additional concern is the different sociodemographic pattern observed in physical activity participation. A systematic review of the relation between socioeconomic position and physical activity identified higher levels of total leisure-time and moderate and vigorous physical activities in individuals with higher socioeconomic status, compared with those with lower socioeconomic status [4]. Additionally, according to several reports [4–6], women living in socioeconomically disadvantaged neighborhoods are at higher risk of physical inactivity, independent of their individual socioeconomic circumstances, which could lead to more pronounced health and social inequalities. Hence, women living in socioeconomically disadvantaged neighborhoods are an important group to target in order to promote physical activity and, consequently, improve their health.

A number of predictors of physical activity participation in adults have been consistently identified, within each of the following areas: demographic and biological factors, psychological, cognitive and emotional factors, behavioral attributes and skills, social and cultural factors, physical environment factors and physical activity characteristics [7]. Perceived barriers to exercise are one of the psychological features that have been suggested as influencing physical activity participation and adherence. Of particular interest are personal barriers, especially in women, because of the lifestyle challenges they face regarding family responsibilities (e.g. childcare commitments) [8, 9]. Indeed, Brownson and colleagues have found that women more frequently report being tired and having no time available, in part due to their domestic situation, as significant perceived barriers to healthy habits than do men [10]. Moreover, Salmon and colleagues [11] showed that personal barriers such as lack of time, other priorities, work, and family commitments were inversely associated with physical activity behavior in women. Thus, a better understanding of the common personal barriers—including family barriers—faced by women regarding positive physical activity changes will likely foster increased participation if translated to interventions.

To better understand long-term physical activity adherence, researchers have focused on the role of qualitative aspects of motivation, as described by Self-Determination Theory (SDT). Among other features, this framework specifies that people can be intrinsically or extrinsically motivated to regulate their own behavior [12, 13]. Intrinsic motivation is the most autonomous form of behavioral regulation and when intrinsically motivated to regulate their own behavior, individuals adopt a specific behavior largely for the experience of the behavior *per se*. By contrast, when extrinsically motivated, individuals can adopt specific behaviors based on external contingencies, internalized self-judgments, personal importance of its consequences, or because it is completely congruent with the person’s other values [12, 13]. Additionally to the motivational regulatory processes underlying a behavior—the “why” of motivation—SDT also emphasizes the contents of individuals’ goals or aspirations for a specific domain of behavior—the “what” of motivation, i.e., the outcomes that individuals are pursuing by engaging in the behavior—which can have intrinsic or extrinsic qualities [14].

A comprehensive review of the literature on the motivational features related with exercise and physical activity outcomes identified an autonomous regulatory style (including intrinsic motivation) as one of the most important factors to foster positive, meaningful, and long-lasting physical activity behavior changes [15]. Also, the pursuit of intrinsic (e.g., seeking affiliation or challenge) rather than extrinsic (e.g., seeking social recognition or appearance improvement) *exercise goals* was shown by Sebire et al. [16] to be positively associated with psychological need satisfaction in exercise, physical self-worth, psychological well-being, and self-reported exercise behavior; and by Vansteenkiste et al. [17] to have a positive effect on effort expenditure, performance, and long-term exercise persistence. Therefore, understanding the motivational dynamics of adopting a physically active lifestyle and especially sustaining it over time is a critical issue and is highly relevant for the development of physical activity interventions with lasting effects [18, 19].

Although there is a growing body of studies providing insights on the relation between these features and physical activity participation and adherence, there is a lack of studies examining such relations in socioeconomically disadvantaged populations. This study contributes to fulfill this gap by examining cross-sectional and longitudinal associations of different aspects of physical activity motivation and perceived barriers with physical activity behavior in women living in socioeconomic disadvantage. In particular, we sought to study the association of i) intrinsic motivation; ii) physical activity goals, namely health, fitness, appearance, weight, relaxation and stress relief goals; and iii) perceived family barriers with short- and long-term leisure-time physical activity (LTPA). Also, because motivation and actual engagement in lifestyle behaviors may differ between those who have and do not have concerns regarding their weight [20], moderating effects of weight control intentions were also tested for the main associations. Importantly, we adjusted all analyses for important facets of the social and physical environment, namely the neighborhood personal safety and walking environment, since the surrounding areas where individuals live—e.g., presence of sidewalks/footpaths, proximal physical activity facilities and street lighting—may influence their choices related to physical activity in their leisure time [21].

## Methods

The present study used data collected in 2007–08 (baseline) and 2010–2011 (three-year follow-up) as part of the Resilience for Eating and Activity Despite Inequality (READI) study, a longitudinal cohort study examining eating behaviors, physical activity and obesity in women and children living in socioeconomically disadvantaged neighborhoods in Victoria, Australia. The READI study was approved by the Deakin University Human Research Ethics Committee, the Victorian Department of Education, and the Catholic Education Office. All participants gave written consent to participate. A detailed description of the study is available elsewhere [22] and will only be summarized here.

### Participants

Women aged between 18 and 46 years were randomly selected using the electoral roll (voting is compulsory in Australia) from 40 rural and 40 urban suburbs of Victoria identified as socioeconomically disadvantage according to the Australian Bureau of Statistics’ 2001 Index of Relative Socio-economic Disadvantage (or SEIFA) [23]. The lifespan under target reflects women of childbearing age, because of their relatively high risk for unhealthy weight gain during this life stage. The sampling source (Australian Electoral Commission) provides samples in age strata and 18–45 years was the closest match to ‘childbearing age’ possible within these strata. By the time women completed the survey, a small number had turned 46. A total of 11940 women were identified (150 women from each of the 80 areas; some included areas had <150 eligible women). From 4934 women (45%, excluding from the denominator those ineligible) who responded to a postal invitation to complete a questionnaire, 571 moved from the sampled neighborhood before completing the survey, 3 were not intended participants, 2 withdrew from the study and 9 did not meet the age range criteria. Thus, data from these women were excluded, leaving 4349 eligible participants at baseline. Of those, 1913 women completed three-year follow-up assessments. Additional details about the sampling and dropout in the READI study have already been reported [22].

Women who were pregnant (n = 162) or who were trying to gain weight (n = 57) at baseline or follow-up were excluded from analyses. Women were also excluded if they were missing data on any of these variables (n = 58). Some women met more than one exclusion criteria, leaving a final sample of 1664 women.

### Covariates

#### Sociodemographic factors

At baseline, women were asked to provide information about their sociodemographic characteristics including age, country of birth (categorized as “Australia” or “other”), highest education level (low—did not complete year 12; medium—completed year 12 or equivalent; or high—tertiary qualification), marital status (married/de facto union, previously married or never married), number of dependent children (none, one, two or three or more), employment status (working full time, working part-time or not working), personal income (categorized as low—$0–299 per week; medium—$300–699 per week; or high—$700+ per week) and household income (categorized as low—$0–699 per week; medium—$700–1499 per week; or high—$1500+ per week).

#### Physical and social environmental factors

Two environment-related variables were assessed at baseline (see Ball et al., 2012 [24] for full details). The neighborhood “personal safety” was assessed by responses to the following three items: “I feel safe walking in my neighborhood day or night”, “Violence is not a problem in my neighborhood”, and “My neighborhood is safe from crime” (Cronbach’s α = 0.85). The neighborhood “walking environment” was evaluated by responses to the following seven statements: “My neighborhood offers many opportunities to be physically active”, “Local sports clubs and other facilities in my neighborhood offer many opportunities to get exercise”, “It is pleasant to walk in my neighborhood”, “The trees in my neighborhood provide enough shade”, “In my neighborhood it is easy to walk places”, “I often see other people walking in my neighborhood”, and “I often see other people exercising (e.g., jogging, bicycling, playing sports) in my neighborhood” (Cronbach’s α = 0.81). Responses to each item were scored in a Likert-type scale ranging from 1 (strongly disagree) to 5 (strongly agree) and a total score for each construct was created through the sum of the respective items.

### Predictors

Eight predictor variables were selected from the baseline data in relation to physical activity participation: intrinsic motivation; health goals, fitness goals, appearance goals, weight goals, relaxation goals, stress relief goals; and perceived family barriers.

Intrinsic motivation was assessed with the following items reflecting feelings in relation to physical activity: “I enjoy it vs. I hate it”, “I feel interested vs. I feel bored”, “I find it pleasurable vs. I find it unpleasurable”, “I find it energizing vs. I find it tiring”, “It makes me happy vs. It makes me depressed”, and “I feel good physically while doing it vs. I feel bad physically while doing it” (Cronbach’s α = 0.95). Answers to the six items were rated on a 7-points Likert scale ranging from 1 (most negative feeling) to 7 (most positive feeling) and a total score was calculated by summing the items.

For physical activity goals assessment, participants rated the importance of each of the following six outcomes of being physically active on a 4-point Likert scale, ranging from 1 (no reason at all) to 4 (a very important reason): “Health”, “Feeling fit”, “Appearance”, “Weight”, “Relaxation” and “Stress relief”.

Perceived family barriers was assessed by responses to the following three items: “I feel guilty doing physical activity when I have family commitments”, “My family commitments usually take priority over my physical activity”, and “I make time for physical activity even when I am busy with family commitments” (Cronbach’s α = 0.79). Responses to each item were scored on a Likert scale ranging from 1 (strongly disagree) to 5 (strongly agree). The responses to the first two items were recoded in the opposite direction and responses were then summed.

### Moderator

Weight-control intention was assessed at baseline by asking women “Which of the following best describes you at the moment?”, and the categories of response were: (1) I am actively doing things to gain weight at the moment; (2) I am actively doing things to try to avoid gaining weight at the moment; (3) I am actively doing things to try to lose weight at the moment; and (4) I am not doing anything in particular for my weight at the moment. Only a small proportion of women (<3%) reported trying to gain weight and therefore they were excluded from analyses since we were primarily interested in the role of weight loss/maintenance intention as a moderator. This variable was recoded in two categories: (1) no weight-control intention and (2) trying to avoid gaining weight or to lose weight.

### Outcome Measure

LTPA at baseline and follow-up was assessed through the long version of the self-administered International Physical Activity Questionnaire (IPAQ-L), a well-established instrument for cross-nationally monitoring population levels of physical activity and inactivity. This questionnaire is suitable for use in adults, has excellent test-retest reliability (pooled r = 0.81) and acceptable validity (mean ρ = 0.30) [25]. Women self-reported time (minutes per week) spent in walking and in moderate- and vigorous-intensity physical activity in their leisure time during the 7 days preceding the assessment, which was summed into a single variable.

### Statistical Analyses

Statistical analyses were carried out using IBM SPSS for Windows, version 21.0. For univariate associations, the significance level was set at *p* ≤ 0.05, corresponding to a 95% Confidence Interval (CI). For multiple linear regression analyses, the significance level was set at *p* ≤ 0.01, corresponding to a 99% CI, to partially correct for the inflated type I error associated with multiple testing. A mean difference or a regression coefficient was considered statistically significant if its 95% or 99% CI did not include zero. Internal consistency estimates were calculated for the two physical environmental constructs, intrinsic motivation and perceived family barriers. Descriptive statistics were conducted, using baseline data, to characterize the sample. Independent-sample *t* tests for age and chi-square tests for categorical variables were used to compare differences between women who were trying to control weight and women who were not trying to control weight. The variable LTPA at baseline and follow-up was non-normally distributed, so logarithmic transformations (natural logarithm) were applied before inclusion in analyses.

Univariate associations of all sociodemographic and a priori selected physical and social environmental variables with LTPA at baseline were examined using linear regression models. All of those variables significantly associated with LTPA at baseline (highest education level, number of dependent children, employment status, personal income, household income and neighborhood “walking environment”) were used as covariates in multiple linear regression models, which were conducted to assess the association between each motivational variable and perceived family barriers and the dependent measure. Subsequently, for each significant association, moderating effects were examined by additionally including the main effect of the weight control intentions (the moderator) into the linear regression models, followed by the interaction between each of the independent variables and weight control intentions.

For prospective analyses, a similar procedure was developed: linear regression modeling was used to assess the relationship between each of the sociodemographic and physical and social environmental variables and LTPA at follow-up, adjusting for LTPA at baseline; those variables significantly associated with the outcome (number of dependent children, employment status and personal income) were then included as covariates in subsequent multiple linear regressions, which were derived to evaluate the association between each motivational variable and perceived family barriers and LTPA at follow-up, always controlling for LTPA at baseline; additionally, for each significant association, the potential moderator (weight control intentions) and the interaction between each of the independent variables and the moderator were introduced in the linear regression models.

If there was a significant moderator effect in either cross-sectional (which was the case for weight goals) or prospective analyses (which was verified for intrinsic motivation), the sample was stratified by trying to control weight/no weight-control intentions and linear regressions models were performed to examine associations between the independent and dependent variables within each group.

## Results

The mean age of women was 36.4 (s.d. = 7.7) years. The majority was born in Australia (92.2%) and was married (70.4%). About one third of the women had no dependent children, 48.5% had medium level of education, 36.5% had a full time job and 36.8% and 49.0% had medium personal and household income, respectively. Compared with women who were not trying to control their weight, women who were trying to lose or maintain weight were more likely to be highly educated (X^2^ = 7.568, 95% CI = 0.019, 0.025), unmarried (X^2^ = 6.797, 95% CI = 0.027, 0.034) and had higher household income (X^2^ = 6.786, 95% CI = 0.031, 0.038). There were no between-group differences with respect to age, country of birth, number of dependent children, employment status and personal income.

Associations of sociodemographic and physical and social environmental factors with LTPA at baseline and follow-up are displayed in Table 1. Regarding sociodemographic factors, highest educational level, employment status, personal income and household income were positively associated with LTPA at baseline and employment status and personal income were positively associated with LTPA at follow-up regardless of baseline LTPA, while number of dependent children had a negative association with both LTPA at baseline and follow-up. Of the physical and social environmental factors, only neighborhood “walking environment” was positively associated with LTPA at baseline and none had significant associations with LTPA at follow-up.

**Table 1:** Univariate associations of sociodemographic and physical and social environmental factors with leisure-time physical activity.

	Leisure-time physical activity at baseline		Leisure-time physical activity at follow-up^1^	
Variables	Standardized β (95% CI)		Standardized β (95% CI)	
**Sociodemographic factors**				
Age	-0.045	(-0.094, 0.004)	0.001	(-0.053, 0.054)
Country of birth	0.032	(-0.017, 0.082)	-0.010	(-0.065, 0.045)
*Highest education level (ref*. = *low*)				
Medium	**0.133**	(0.072, 0.195)	-0.041	(-0.113, 0.032)
High	**0.213**	(0.151, 0.274)	-0.037	(-0.108, 0.034)
*Marital status (ref*. = *never married*)				
Married / de facto	-0.049	(-0.104, 0.006)	-0.048	(-0.108, 0.013)
Previously married	-0.037	(-0.093, 0.018)	-0.023	(-0.085, 0.038)
*Number of dependent children (ref*. = *none*)				
One	**-0.082**	(-0.137, -0.027)	**-0.068**	(-0.131, -0.008)
Two	**-0.084**	(-0.140, -0.028)	-0.019	(-0.081, 0.043)
Three or more	**-0.060**	(-0.114, -0.005)	0.036	(-0.025, 0.097)
*Employment status (ref*. = *not working*)				
Working full time	**0.070**	(0.012, 0.128)	**0.085**	(0.019, 0.148)
Working part-time	**0.066**	(0.008, 0.125)	0.006	(-0.059, 0.071)
*Personal income (ref*. = *low*)				
Medium	0.040	(-0.019, 0.099)	0.020	(-0.047, 0.088)
High	**0.099**	(0.039, 0.157)	**0.081**	(0.014, 0.145)
*Household income (ref*. = *low*)				
Medium	**0.114**	(0.037, 0.186)	0.040	(-0.047, 0.127)
High	**0.156**	(0.077, 0.226)	0.050	(-0.036, 0.135)
**Physical and social environmental factors**				
Neighborhood “personal safety”	0.045	(-0.004, 0.094)	-0.022	(-0.077, 0.033)
Neighborhood “walking environment”	**0.163**	(0.115, 0.212)	0.032	(-0.023, 0.088)

Statistical significance is represented in bold type.

^1^ adjusted for leisure-time physical activity at baseline

Table 2 shows associations of motivational variables and perceived family barriers at baseline with LTPA at baseline, and the moderating effects of weight control intentions. All of the predictor variables, including weight control intentions, showed positive associations with the dependent variable (model A). However, results of the moderated regression analyses (model B) indicated that at baseline, weight control intentions only had a moderator effect in the associations between weight goals for being physically active and LTPA (β = 0.538, 99% CI = 0.057, 0.990). When the sample was stratified by weight control intentions, weight goals were not significantly associated with LTPA at baseline for either women actively trying to control their weight (β = 0.078, 99% CI = -0.021, 0.194) and women who were not trying to control their weight (β = -0.103, 99% CI = -0.228, 0.038).

**Table 2:** Associations between physical activity motivation and barriers and leisure-time physical activity at baseline, and moderator effects of weight control intentions.

	Model A^1^		Model B^2^					
	Main effect of independent variable		Main effect of independent variable		Main effect of moderator (weight control intentions)		Interactions (independent variable x moderator)	
Intrinsic motivation	**0.380**	(0.295, 0.447)	**0.337**	(0.069, 0.589)	0.152	(-0.154, 0.452)	0.020	(-0.406, 0.444)
Health goals	**0.082**	(0.001, 0.166)	-0.027	(-0.293, 0.238)	0.083	(-0.378, 0.540)	0.171	(-0.386, 0.718)
Fitness goals	**0.128**	(0.048, 0.209)	-0.056	(-0.336, 0.224)	0.023	(-0.328, 0.373)	0.270	(-0.208, 0.735)
Appearance goals	**0.124**	(0.044, 0.199)	-0.029	(-0.323, 0.266)	0.128	(-0.146, 0.394)	0.145	(-0.296, 0.576)
Weight goals	**0.084**	(0.004, 0.162)	**-0.286**	(-0.550, -0.017)	-0.123	(-0.439, 0.199)	**0.538**	(0.057, 0.990)
Relaxation goals	**0.134**	(0.053, 0.210)	0.170	(-0.123, 0.456)	**0.266**	(0.015, 0.504)	-0.065	(-0.439, 0.314)
Stress relief goals	**0.106**	(0.026, 0.184)	0.053	(-0.236, 0.340)	0.191	(-0.069, 0.443)	0.054	(-0.335, 0.439)
Perceived family barriers	**0.202**	(0.123, 0.293)	0.118	(-0.188, 0.430)	0.170	(-0.037, 0.370)	0.079	(-0.302, 0.464)

Data reported as Standardized β with 99% Confidence Intervals to partially correct for the inflated type I error associated with multiple testing (statistical significance is represented in bold type).

^1^Associations between each independent variable and the dependent variable, adjusted for highest education level, number of dependent children, employment status, personal income, household income and neighborhood “walking environment”

^2^ Interactions between each independent variable and the moderator, adjusted for highest education level, number of dependent children, employment status, personal income, household income and neighborhood “walking environment”

**Fig 1:**
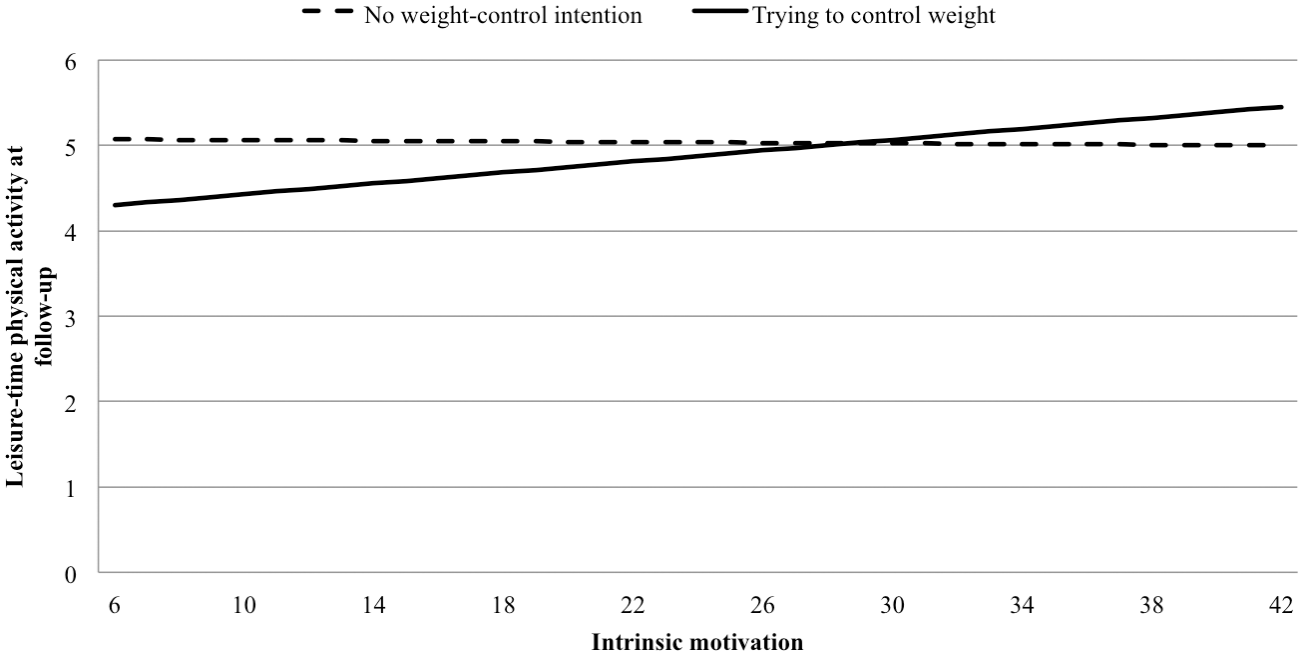
Associations between intrinsic motivation and leisure-time physical activity at follow-up among women trying vs. not trying to control their weight. Note: plots show associations when leisure-time physical activity at baseline was fixed at the geometric mean, number of dependent children was fixed to two, employment status was fixed to working full time and personal income was fixed to medium.

Table 3 presents associations of motivational variables and perceived family barriers at baseline with LTPA at follow-up, controlling for LTPA at baseline, and moderator effects of weight control intentions. Intrinsic motivation, appearance and relaxation goals for being physically active and weight control intention showed positive associations with LTPA at follow-up (model A). Moderated regression analyses (model B) revealed that weight control intentions only significantly moderated the association between intrinsic motivation and LTPA at follow-up (β = 0.666, 99% CI = 0.188, 1.145). When stratified by weight control intentions (Fig 1), intrinsic motivation was significantly associated with LTPA at follow-up for women actively trying to lose or maintain weight (β = 0.184, 99% CI = 0.097, 0.313), but not for women with no weight-control intentions (β = -0.011, 99% CI = -0.159, 0.139).

**Table 3:** Associations between physical activity motivation and barriers and leisure-time physical activity at follow-up, and moderator effects of weight control intentions.

	Model A^1^		Model B^2^					
	Main effect of independent variable		Main effect of independent variable		Main effect of moderator (weight control intentions)		Interactions (independent variable x moderator)	
Intrinsic motivation	**0.140**	(0.060, 0.234)	-0.251	(-0.564, 0.036)	**-0.381**	(-0.737, -0.032)	**0.666**	(0.188, 1.145)
Health goals	0.042	(-0.037, 0.125)						
Fitness goals	0.061	(0.017, 0.142)						
Appearance goals	**0.078**	(0.000, 0.154)	-0.178	(-0.478, 0.125)	-0.114	(-0.389, 0.158)	0.356	(-0.090, 0.793)
Weight goals	0.044	(-0.033, 0.123)						
Relaxation goals	**0.097**	(0.018, 0.175)	-0.040	(-0.331, 0.252)	-0.008	(-0.258, 0.242)	0.183	(-0.198, 0.560)
Stress relief goals	0.077	(-0.001, 0.153)						
Perceived family barriers	0.046	(-0.036, 0.126)						

Data reported as Standardized β with 99% Confidence Intervals to partially correct for the inflated type I error associated with multiple testing (statistical significance is represented in bold type).

^1^Associations between each independent variable and the dependent variable, adjusted for leisure-time physical activity at baseline, number of dependent children, employment status and personal income

^2^ Interactions between each independent variable and the moderator, adjusted for leisure-time physical activity at baseline, number of dependent children, employment status and personal income

**Fig 2:**
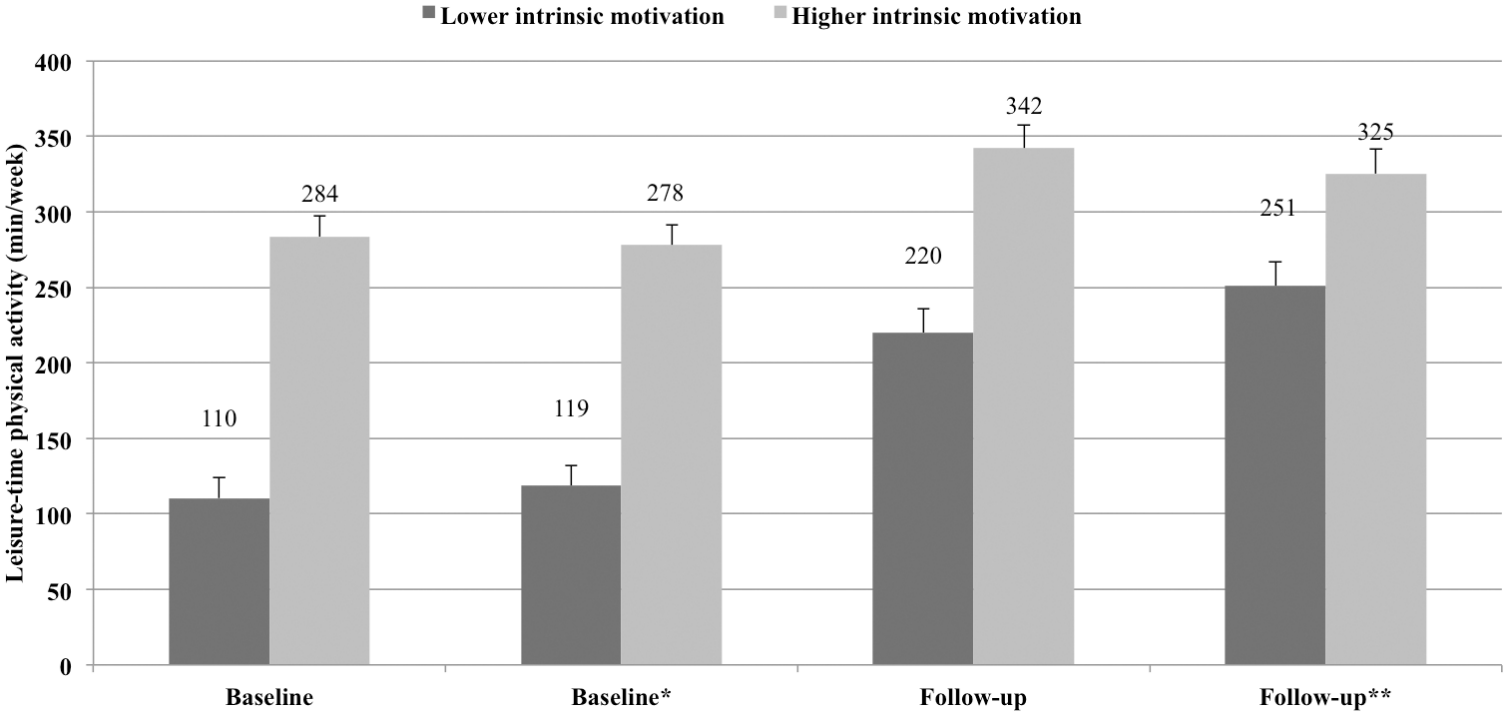
Associations between intrinsic motivation and leisure-time physical activity at baseline and at follow-up (between-group comparisons of estimated marginal means with linearly independent pairwise tests: baseline—mean dif. = -173 min/week, 99% CI = -223, -124; baseline*—mean dif. = -159 min/week, 99% CI = -209, -110; follow-up—mean dif. = -122 min/week, 99% CI = -175, -69; follow-up**—mean dif. = -74 min/week, 99% CI = -131, -18). Note: Groups represent the lowest and highest tertile-split groups of adjusted intrinsic motivation means at baseline. At baseline, adjustments were made for highest education level, number of dependent children, employment status, personal income, household income and neighborhood “walking environment” (*and weight control intentions); at follow-up, adjustments were made for number of dependent children, employment status and personal income (**and leisure-time physical activity at baseline and weight control intentions). Error bars indicate the standard error of the mean.

For a more specific analysis of how intrinsic motivation related to LTPA, two groups representing the first (lowest) and third (highest) tertiles of adjusted intrinsic motivation means at baseline were created. Fig 2 shows associations between these groups and LTPA at baseline and follow-up (controlling and not controlling for weight control intentions and LTPA at baseline). At follow-up, LTPA participation was higher for both groups of intrinsic motivation and the group displaying higher intrinsic motivation showed higher participation in LTPA at both baseline and follow-up.

## Discussion

Socioeconomically disadvantaged individuals are at higher risk of physical inactivity [4–6] and have been understudied concerning physical activity correlates. To our knowledge, this is the first study to examine cross-sectional and prospective associations of motivational factors and perceived family barriers with physical activity behavior in a large sample of women living in socioeconomically disadvantaged communities. As expected, results showed that all motivational variables and perceived family barriers were correlated with LTPA at baseline, even after adjusting for sociodemographic and physical and social environmental factors. Across analyses, intrinsic motivation was consistently associated with LTPA (independently of significant sociodemographic factors and also baseline values of LTPA in prospective analysis), suggesting, in line with SDT, that the perception of enjoyment, interest and challenge present when engaging in the behavior are important driving forces of sustained behavioral adherence [15]. Regarding goals for being physically active, relaxation goals were consistently associated with physical activity participation (independently of significant covariates), although less strongly at follow-up.

Results from this study are in agreement with prior research showing a consistent positive association of intrinsic motivation and more intrinsic goals with exercise [15]. Although stress relief goals are more intrinsically-oriented, they did not predict physical activity participation at follow-up. Appearance goals were also associated with physical activity participation (also less strongly at follow-up), although mixed associations of body-related goals with exercise have been observed in other studies [15]. Indeed, according to SDT, some goals can have both intrinsic and extrinsic motivational features, depending on the level of autonomy (or personal endorsement) involved in choosing that behavior. Individuals with more self-determined motivation can sustain adherence to a particular behavior (in this case, LTPA) even holding extrinsic goals (e.g., improving appearance) [26]. Finally, weight, fitness, and health goals did not predict LTPA at follow-up. According to Markland and Ingledew [26], weight-related goals tend to be experienced as controlling and so contribute little to long-term physical activity participation, although mixed results are reported across studies. Supplementary exploratory factor analysis including the six items (data not shown) revealed weight goals grouped in a latent construct with appearance goals (Cronbach’s α = 0.71), suggesting a more extrinsic nature. Similarly, the literature also shows mixed results for fitness and health goals, possibly because they can reflect either more controlling or more autonomous reasons like pursuing a socially-valued body image or health pressures exerted by the medical doctor *versus* increasing physical function and competence or a genuine desire for health improvement, respectively [15].

The fact that this population faces socioeconomic disadvantage—which may pose several challenges to daily life, including financial, social and environmental stressors—can partially explain the non-significant results for fitness and health goals and physical activity participation at follow-up, as these goals may assume relatively less importance to the short-term subsistence or to other pressing priorities in this kind of populations. Health is a generalized concept, it has more expression in the presence of sickness, and it can involve other factors related with stress and well-being. Thus, the health-related goals expressed initially (including being physically healthy) may fade away in time and be replaced by other (more valued) goals linked with relaxation and enjoyment associated with long-term physical activity participation. This can also be applied to understand perceived family barriers. In the long run, people may experience feelings of mastery and efficacy in overcoming perceived barriers and so perceived family barriers may become less important or disappear. In fact, in the context of a cognitive-behavior weight loss intervention, Linde *et al*. showed that self-efficacy for exercise behaviors (which is closely related with perceived barriers for exercise) was significantly associated with exercise behaviors and with weight loss monitoring behaviors, such as days of tracking adherence to physical activity plans during the treatment period, but not during follow-up [27].

By conducting analyses that examined motivational variables and perceived family barriers as main correlates of physical activity, and weight-control intention as a moderator of these relations, we were able to examine specifically whether these associations differed in women who were trying *versus* not trying to control weight. Since a large proportion of adults, worldwide, report that they are trying to control their weight [28–31], including women living in socioeconomic disadvantage [32], addressing this issue is particularly important for informing the development of interventions that are appropriately tailored to the population of interest. Weight-control intention moderated the association between weight goals for being physically active and LTPA at baseline, suggesting that establishing weight-related goals and trying to control weight simultaneously predicts physical activity participation (at least cross-sectionally). In prospective analyses, weight-control intention moderated the association between intrinsic motivation and LTPA. When the sample was stratified by weight control intentions, we observed that intrinsic motivation was associated with LTPA at follow-up, irrespective of baseline LTPA, for women actively trying to control their weight at baseline. These results suggest that especially in women initially trying to lose and maintain weight, finding a source of intrinsic motivation may be critical in the longer term. These findings are consistent with results from a weight management intervention showing that, in overweight and obese women who underwent to a 1-year SDT-based intervention, not all types of motivation predicted physical activity and weight loss maintenance; intrinsic and autonomous motivation were the only predictors of long-term outcomes [33, 34].

Strengths of the study include the longitudinal prospective design; the large sample of women living in socioeconomically disadvantaged neighborhoods, a high risk population group for overweight and other health problems, difficult to reach in research studies and interventions; and the wide variety of potential sociodemographic and physical and social environment confounders in physical activity behavior. To our knowledge, very few physical activity long-term participation studies have controlled for a strong set of environmental variables.

This study has also limitations, namely the relatively low response rate (45%), which possibly makes the sample less representative of the women living in socioeconomically disadvantaged neighborhoods—comparing with the general population of women living in the 80 neighborhoods, a greater proportion of READI women was Australian born and was married or living as married, but a lower proportion was in full-time employment [22]–and perhaps show a selection bias towards more motivated women; the limited age range (18–46 years) of the women, making results not generalizable to other age groups of this particular population; and the self-report nature of all constructs, particularly LTPA, which can be affected by recall difficulties and possible overestimations of frequency and duration [35]. Future studies should include objective measurements, such as accelerometry. Another limitation relates to the scales used to assess physical activity motivation and goals. This study was part of a larger study—the READI study—and the definition of the assessments was established prior to the current analysis; therefore, motivational constructs were not assessed with standardized instruments. However, the items used in each construct are very similar to the ones included in validated and commonly used SDT-based instruments (e.g., the Intrinsic Motivation Inventory [36]) and showed good internal consistency. Also, physical activity goals were assessed with single-item measures, which is less than ideal. Although multiple-item scales tend to be more reliable in psychometric assessment and ensure content validity, a single item may be adequate when the construct is highly schematized for most individuals, reflects subjective experience, and when the content of the construct is unidimensional [37] as in this case. Bergkvist and Rossiter reported empirical findings indicating that both single-item and multiple-item measures had equally high predictive validity [38, 39].

Finally, the prospective nature of the analyses included in this study is less than sufficient for causality between baseline predictors and follow-up measures to be inferred. Results should only be interpreted as suggestive and supportive of the hypotheses that the significant prospective correlates may indeed exert effects over time on the outcomes of interest.

## Conclusions

The present findings suggest that disadvantaged women who are attempting to lose or maintain weight and also report high levels of intrinsic motivation may be more protected regarding their LTPA levels over time. Also, in this particular population, weight-related physical activity goals seem to play a role regarding short-term LTPA participation. Based on the current results, weight loss interventions focusing on promoting intrinsic motivation may be especially effective to result in LTPA adherence in this specific population subgroup.
